# In-hospital risk stratification algorithm of Asian elderly patients

**DOI:** 10.1038/s41598-022-18839-9

**Published:** 2022-10-20

**Authors:** Sazzli Kasim, Sorayya Malek, Song Cheen, Muhammad Shahreeza Safiruz, Wan Azman Wan Ahmad, Khairul Shafiq Ibrahim, Firdaus Aziz, Kazuaki Negishi, Nurulain Ibrahim

**Affiliations:** 1 Cardiology Department, Faculty of Medicine, Universiti Teknologi MARA (UiTM), Shah Alam, Malaysia; 2 Bioinformatics Division, Institute of Biological Sciences, Faculty of Science, University of Malaya, Kuala Lumpur, Malaysia; 3 Cardiac Vascular and Lung Research Institute, Universiti Teknologi MARA (UiTM), Shah Alam, Malaysia; 4 National Heart Association of Malaysia, Heart House, Kuala Lumpur, Malaysia; 5 Division of Cardiology, University Malaya Medical Centre, Kuala Lumpur, Malaysia; 6 Department of Artificial Intelligence, Faculty of Computer Science and Information Technology, University of Malaya, Kuala Lumpur, Malaysia; 7 Sydney Medical School Nepean, Faculty of Medicine and Health, Charles Perkins Centre Nepean, The University of Sydney, Sydney, NSW Australia; 8 Nepean Hospital, Sydney, NSW Australia; 9 Faculty of Medicine, Universiti Teknologi MARA (UiTM), Sungai Buloh Campus, Sungai Buloh, Malaysia

**Keywords:** Computational biology and bioinformatics, Cardiology

## Abstract

Limited research has been conducted in Asian elderly patients (aged 65 years and above) for in-hospital mortality prediction after an ST-segment elevation myocardial infarction (STEMI) using Deep Learning (DL) and Machine Learning (ML). We used DL and ML to predict in-hospital mortality in Asian elderly STEMI patients and compared it to a conventional risk score for myocardial infraction outcomes. Malaysia's National Cardiovascular Disease Registry comprises an ethnically diverse Asian elderly population (3991 patients). 50 variables helped in establishing the in-hospital death prediction model. The TIMI score was used to predict mortality using DL and feature selection methods from ML algorithms. The main performance metric was the area under the receiver operating characteristic curve (AUC). The DL and ML model constructed using ML feature selection outperforms the conventional risk scoring score, TIMI (AUC 0.75). DL built from ML features (AUC ranging from 0.93 to 0.95) outscored DL built from all features (AUC 0.93). The TIMI score underestimates mortality in the elderly. TIMI predicts 18.4% higher mortality than the DL algorithm (44.7%). All ML feature selection algorithms identify age, fasting blood glucose, heart rate, Killip class, oral hypoglycemic agent, systolic blood pressure, and total cholesterol as common predictors of mortality in the elderly. In a multi-ethnic population, DL outperformed the TIMI risk score in classifying elderly STEMI patients. ML improves death prediction by identifying separate characteristics in older Asian populations. Continuous testing and validation will improve future risk classification, management, and results.

## Introduction

Acute coronary syndrome (ACS) is the world's leading cause of death and the leading cause of morbidity and mortality in the elderly^1–3^. In the majority of developing countries, the elderly are defined as individuals over the age of 65^4^. Age is a significant risk factor for ACS, and the prevalence of elderly patients presenting with ST-elevation myocardial infarction (STEMI) is increasing in developing countries due to an ageing population^5,6^. Elderly patients have a higher mortality rate, due to more comorbidities and were less likely to get evidence-based treatments^7–9^. With the advancement of general healthcare, elderly are likely to account for a significant proportion of all ACS patients in the future^6^. However, limited data are available on the delivery of health care and clinical outcomes of elderly patients with cardiovascular disease in the South-East Asia region. Elderly patients with Acute Coronary Syndrome (ACS) are also poorly analyzed and underrepresented in modern-day ACS trials^10^.

Common scoring systems such as Thrombolysis in Myocardial Infarction (TIMI) and Global Registry of Acute Coronary Events (GRACE) risk scores are often used to predict mortality for elderly patients^11,12^. TIMI and GRACE scores were developed to predict short-term prognoses based on patients mainly from countries in North America, South America, and Europe, with only Australia and New Zealand providing data from Asian countries to the GRACE registry, despite Asia hosting 60% of the world’s population^13^.

With the current advances and success of deep learning (DL) and machine learning (ML) algorithms such as random forest (RF), extreme gradient boosting (XGB), logistic regression (LR), and Support Vector Machine (SVM) in ACS mortality prediction over conventional risk scores, these algorithms have been adopted for clinical predictions^13–18^. In comparison to DL, ML algorithms require feature selection to attain higher performance accuracy^19,20^. DL algorithms allow automatic learning of the feature and relationship from a dataset minus the necessity for feature selection and attained higher accuracy than ML for mortality prediction. However, unlike ML algorithms, the interpretation of the significant factors for determining risk scores in DL models is unknown^13^.

There has been no research reported on integrating DL with ML feature selection to better understand DL's "black box" feature selection characteristic. Identifying features associated with mortality in the Asian elderly is essential for better patient management in clinical practice. We hypothesize that integrating DL with ML feature selection algorithms will improve in-hospital mortality prediction in Asian elderly STEMI patients. This is an objective, should also clarify that it is a first in world study!

As a result, we propose to integrate ML feature selection with a DL classification algorithm for the prediction and identification of factors associated with in-hospital mortality in multiethnic elderly Asian patients admitted with STEMI. Apart from that, we aim to evaluate the performance of ML with that of DL developed using both complete and selected features from the ML feature selection technique. Additionally, the developed ML and DL prediction models will be compared to the TIMI risk score, which is calculated from multi-ethnic registry data on Asian elderly STEMI patients.

## Materials and methods

### Study population

We examined data from the Malaysian National Cardiovascular Disease Acute Coronary Syndrome (NCVD-ACS) registry from 2006 to 2017 on 17, 227 in-hospital STEMI patients, 3991 of whom were elderly (65 years and above). The raw data used in this study was approved and granted permission to access study data from the National Heart Association of Malaysia (NHAM).

NCVD informed patient consent was waived where for each patient treated at one of the participating hospitals, the registry collects data on a defined set of clinical, demographic, and procedural information^21,22^. The UiTM ethics committee (Reference number: 600-TNCPI (5/1/6)) and the National Heart Association of Malaysia (NHAM) also authorized the study. The ethic approval for NCVD ACS have been applied by the principal investigator of each participating institution and have been approved by Malaysian Research Ethic Committee (NMRR: 07-38-164). The data utilised in this study were anonymized prior to usage, as our study data are interested in the values and parameters without accessing patient personal information.

All patients aged 65 years and above from the registry without exclusion were used including patients who received reperfusion (fibrinolysis, primary PCI (PPCI), angiography demonstrating spontaneous reperfusion, or urgent coronary artery bypass grafting (CABG)) for STEMI. STEMI was characterized as persistent ST-segment elevation ≥ 1 mm in two contiguous electrocardiographic leads, or the presence of a new left bundle branch block in the setting of positive cardiac markers. Input variables are features that are used as input in the development of a model to predict the outcome (in-hospital mortality). To develop the initial model in this study, 50 input variables (9 continuous, 41 categorical) representing columns of patient data from the NCVD data registry were used. The fifty variables used in this study are listed in Table 1. Variables used for model development are variables in the emergency department as first contact as well as variables in the hospital. Follow-up variables were excluded from the analysis. Supplementary table 1 shows the missing rates for each variable used in this study.

**Table 1:** Hyperparameters used for all the DL models.

Hyper parameters	Tuning values				
	DL (all)	DL (LR selected)	DL (RF selected)	DL (SVM selected)	DL (XGBoost selected)
	51	15	13	11	6
Numbers of nodes	277 (51,128,62,32,2)	(15,32,16,8,2)	(13,32,16,8,2)	(11,32,16,8,2)	(6,16,8,4,2)
Numbers of hidden layers	3	3	3	3	3
Drop-out rate	0.2	0.2	0.2	0.2	0.2
Learning rate	0.01	0.01	0.01	0.01	0.01
Optimizer	Adagrad	Adagrad	Adagrad	Adagrad	Adam
Epoch	200	200	200	200	100
Batch size	16	16	16	16	16
Activation function	ReLU	ReLU	ReLU	ReLU	ReLU
					

Categories of variables used are; sociodemographic characteristics, CVD diagnosis and severity, CVD risk factors, CVD comorbidities, non-CVD comorbidities, clinical presentation, baseline investigation, electrocardiography, treatments, and pharmacological therapy. The National Cardiovascular Disease Database (NCVD)—Acute Coronary Syndrome (ACS) registry, which is documented by the National Heart Association of Malaysia, defines the criteria for variables such as hypertension, diabetes, history of heart failure, and chronic renal disease^23^.

For in-hospital mortality, the time frame was calculated from the first hospital admission. Deaths were confirmed yearly through record linkages with the Malaysian National Registration Department. The registry's data does not include information on short-term complications such as heart failure. The follow-up data points are intended to collect these variables, but due to the high number of missing values, we omitted them from the study. To increase the impact of the study, we focused our algorithm on policy-changing hard endpoints such as death. This was done in other publications as well^13,15,24^.

### Complete cases

We have used a complete set of data for primary analysis to ensure the validity of the findings for model development. The primary analysis was performed on complete cases, and the secondary analysis was performed on the top-performing algorithm using missing cases after data imputation.

A total of 3991 in-hospital elderly STEMI patients aged 65 and above were collected from the registry. The final dataset of complete cases of elderly patients of 1345 datasets was identified as complete cases used for primary analysis (with no missing values on predictors). This rendered patients with a full predictor set of 50 variables (9 continuous, 41 categorical) for the study as shown in Table 1.

### Missing cases

Secondary analyses were conducted on the top-performing algorithm after adding 2646 missing cases for a total of 3991 cases. We employed chained equations and predicted mean matching to perform multivariable imputation^25^.

This method imputes missing values based on real values from other cases where predicted values are closest. We used multiple imputations, which means that missing data is typically imputed five times^25^.

Our definition of an incomplete dataset includes variables that are missing up to 30%. There is no missing data for electrocardiography, but there is less than 2% to 10% missing data for demographics, pharmacological therapy, invasive therapeutic procedures, smoking status, smoking history, diabetes, hypertension, and clinical representation such as systolic and diastolic blood pressure. Missing variables are reported to be less than 15% for chronic lung and renal disease, as well as a history of myocardial infarction, heart failure, and cerebrovascular disease. There is 20% missing data for baseline invention variables, and up to 30% missing data for Killip class and heart rate.

The referenced missing dataset is for patient characteristics, not outcome data. Due to the prospective nature of our dataset and the retroactive administration of data, the level of missing values across all variables was completely unpredictable and beyond our control. In our dataset, the likelihood of missing values is independent of both the observed values in any variable and the unseen portion of the dataset.

As a result, the dataset is classed as missing completely at random (MCAR), which indicates that the pattern of missing values is random and not dependent on any variable that may or may not is included in the study.

### Development of risk models

A stratified random sampling of data was used from Kuhn and Johnson study^26^. Data were split for model development (70%) and validation (30%) for all models. Multiple admissions are counted as one for each patient; the splits are based on patient identifiers rather than individual examples. The same pool dataset is assigned to patients with the same identifier. This means that if a patient is admitted three times, each of those three admissions will be assigned to the same set of either training or testing. The patient identifier was replaced with a randomly generated patient identifier to ensure the anonymity of the dataset used in this study^27^.

We accessed the performance of DL and ML algorithms with TIMI using a validation set that accounts for 30% of data that is not used for model development.

Prediction models for the elderly with STEMI were developed using the R package (Version 3.5.2) for DL and conventional ML algorithms such as LR, RF, XGboost, and SVM. These algorithms were selected due to their high performance in previous cardiovascular disease studies. The ML algorithms LR, RF, XGboost, and SVM feature selection methods are used to rank the variables listed in Table 1. Iterative feature selections were performed on the ranked variables in ascending order iteratively to generate the final variables^28^. Cross-validation was used to avoid overfitting for model development on the training set^29^. The ML prediction models were trained and tested for each iteration, and the models with the highest performance were selected. Predictive performances of the models were calculated using the validation dataset. DL models were then constructed with features selected from ML feature selection.

#### Random forest (RF)

RF algorithm implemented in this study was based on Breiman study^30^. Varying value of *entry* and number of trees *ntree* (500–4000) was used in this study to determine the optimum RF model that produced the best results. The RF variable importance method was used to generate ranked variables that were then reduced using sequential backward elimination iteratively. The final model for RF classifier parameters is *ntree* = 1000, and *mtry* = 6.

#### Support vector machine (SVM)

SVM was implemented in this study using the RBF kernel^31^. SVM in this study uses ROC curve variable importance to select and rank the most important variables. The final parameter after tuning used is sigma = 0.01 and c = 0.25 (cost tuning parameter, which regulates the margin width).

#### Logistic regression

The LR model was constructed using the generalized linear model function with family binomial. We used the original Akaike IC as the information criterion and backward directions for the LR model feature selection. LR in this study was constructed using default parameters.

#### XGB

XGB is an implementation of gradient boosting. XGB gives a more accurate result because it used a more regularised form of Gradient Boosting which improves model generalization capabilities that can control overfitting. Besides, it used parallel tree learning which makes the learning process faster. It is more capable of handling missing values compare to gradient boosting^32^. Default parameters have been used for XGB model development in this study.

#### Deep learning

We used a multilayer perceptron (MLP) based on deep learning that integrates four hidden layers, 100–200 nodes, batch normalization, and dropout layers^33–35^. Three hidden layers were used as there is no significant increase in performance when more layers were added. We used the R version of the Tensor Flow and Adam optimizer with the default parameters and binary-cross entropy as the loss function^36^. Rectified linear unit (ReLU) as the activation function^37^ was used after comparing with other activation functions predictive performance such as SoftMax, linear, Tanh, leaky ReLU, and exponential linear unit. The hyper-parameters used in the development of DL were tuned using grid search and manual tuning. Data for DL model development, categorical variable values were replaced with numeric values, and continuous variable values were normalised using z-scores^38^. Data preprocessing was performed in the training data and validation data, separately. Table 1 also covered the hyperparameters that were used in all of the deep learning models.

### Feature selection

The ML algorithms LR, RF, XGboost, and SVM feature selection methods are used to rank the variables listed in Table 1. Sequential Backward Elimination (SBE) algorithm was then applied to the ranked list of variables in ascending order to generate the final variables.

The sequential Backward elimination algorithm relies only on significance as a sufficient condition to remove insignificant variables from a model^39^. Dependencies among variables are considered to obtain better performance^40^. Variables are eliminated in ascending order of importance from RF, XGB, and SVM feature selection methods. The prediction model is retrained and tested each time a variable is eliminated. The variable that causes a decrease in the AUC of the prediction model upon elimination based on the ranked variable list using RF, XGB and SVM feature selection is retained. The retained variables were ranked again using feature importance and the elimination process is repeated until the model with the least number of variables and the highest AUC value is achieved. LR feature selection was done using built feature selection using Akaike IC as the information criterion and backward directions. DL algorithm does not provide built-in feature importance. It has automatic learning of features and relationships from a given data, hence feature importance for the model is unknown. However, we have applied features selected from RF, XGB SVM, and LR to DL model development in this study.

### Model evaluation, validation, and performance measures

The calibration of the models was compared using standardized measures^41^. The area under the curve (AUC) was used as a predictive performance metric. Additional performance metrics were accuracy, sensitivity, specificity, positive predictive value (PPV), and negative predictive value (NPV) for model calibration. Paired resampled t-test was used to compare the ML model’s predictive performances^26^. The net reclassification index (NRI) was also assessed to evaluate the percentage improvement in identifying both positive and negative cases with the best model compared to the TIMI risk score^42^.

### Comparison with conventional method TIMI score

Calculated TIMI scores were used from the NCVD registry for the validation data performance. TIMI score performance (AUC) was compared with the developed DL and ML—models using the validation set that was not used for model development. A graph was also derived to compare performance with the TIMI score based on cutoff points applicable in clinical practice and literature^43^. We define the high risk of death as a probability rate of > 8% similar to that reported by^43^. The ML and DL high-risk population in this study is defined as a mortality probability of > 40% which is equivalent to the TIMI score of > 5.

### Additional statistics

**Figure 1:**
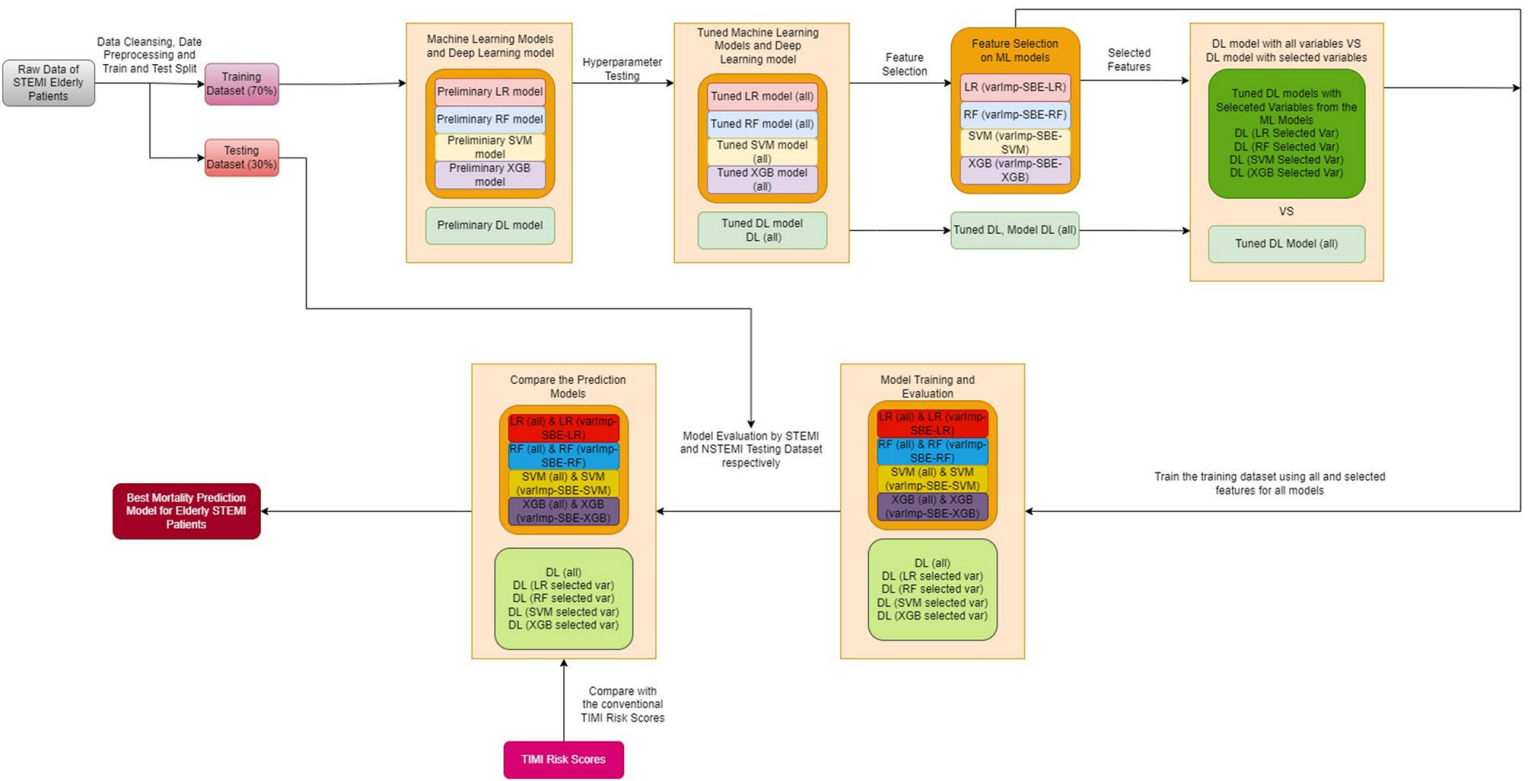
Research workflow and methodology applied in this study.

The results are expressed as mean and SD for continuous variables and as frequencies for categorical variables. Correlation analysis was carried out to identify a significant relationship between variables. Univariate analysis was performed using a Chi-Square test to identify significant variables and a two-sided independent student t-test (p < 0.05). The DL and ML performance was compared using a pair-wise corrected resampled t-test^29,44^. Statistical significance was considered if the p-value was less than 0.0001. Figure 1 summarizes the workflow and methods used in this study.

### Ethical declaration

This study was approved by the UiTM Research Ethics Committee (Reference: 600-TNCPI (5/1/6)), with the approval code REC/673/19. The UiTM Ethics Committee operates in accordance to the ICH Good Clinical Practice Guidelines, Malaysia Good Clinical Practice Guidelines and Declaration of Helsinki.

## Results

### Patient characteristics

Table 2 depicts the summary statistics for the complete set of cases used in the study. The in-hospital complete feature dataset of elderly STEMI patients has a mean age of 72 years. The majority of patients in the dataset are male (74%), Malay (53.9%), non-smokers (46%), and had a history of chronic diseases such as hypertension (69.1%), diabetes (46%), chronic angina (11.1%), myocardial infarction (9.9%), cardiovascular disease (8%), chronic renal disease (7.4%), peripheral vascular disease (5.3%), heart failure (3.0%), and chronic lung disease (2.9%). Percutaneous coronary intervention (PCI) was used to treat approximately 29% of patients. The overall mortality rate of elderly patients is 37%. There was a significant difference between survival and non-survival in age, ethnicity, diabetes, chronic renal disease, heart rate, systolic blood pressure, diastolic blood pressure, Killip classification, total cholesterol, high-density lipoprotein cholesterol, low-density lipoprotein cholesterol, fasting blood sugar, bundle branch block, cardiac catheterization, aspirin, beta-blockers, ACE inhibitor, diuretics where all variables have p-values < 0.0001.

**Table 2:** Summary statistic of complete and imputed dataset.

Predictors (n = 51)	Complete dataset				Imputed dataset			
	All cases (n = 1407)	Survivors (n = 902)	Non-survivors (n = 505)	p-value	All cases (n = 4053)	Survivors (n = 2262)	Non-survivors (n = 1791)	p-value
**Socio-demographic characteristics**								
Age (yrs)*	72.15 ± 5.91	71.17 ± 5.21	73.91 ± 6.63	** < 0.0001**	62.53 ± 23.43	71.89 ± 5.51	73.42 ± 5.94	** < 0.0001**
**Gender**				0.670				
Male	1051 (74.0%)	664 (73.6%)	377 (74.7%)		2739 (67.6%)	1625 (71.8%)	1145 (63.9%)	
Female	366 (26.0%)	238 (26.4%)	128 (25.3%)		1314 (32.4%)	637 (28.2%)	646 (36.1%)	
Ethnicity*				** < 0.0001**				**0.003**
Malay	752 (53.4%)	426 (47.2%)	326 (64.6%)		2226 (54.9%)	1226 (54.2%)	1014 (56.6%)	
Chinese	384 (27.3%)	261 (28.9%)	123 (24.4%)		959 (23.7%)	599 (26.5%)	401 (22.4%)	
Indian	209 (14.9%)	163 (18.1%)	46 (9.1%)		665 (16.4%)	304 (13.4%)	287 (16.0%)	
Others	62 (4.4%)	52 (5.8%)	10 (2.0%)		203 (5.0%)	133 (5.9%)	89 (5.0%)	
**Status before event**								
Smoking status				0.394				0.030
No smoker	659 (46.8%)	425 (47.81%)	234 (46.3%)		1865 (46.0%)	1024 (45.3%)	864 (48.2%)	
Former smoker	346 (25.6%)	210 (23.2%)	136 (26.9%)		1149 (28.3%)	531 (23.5%)	435 (24.3%)	
Current smoker	402 (28.6%)	267 (29.6%)	135 (26.7%)		1039 (25.6%)	707 (31.3%)	492 (27.5%)	
Smoking status (male)				0.203				0.019
No smoker	325 (31.2%)	208 (31.3%)	117 (31.0%)		795 (29.0%)	526 (32.2%)	269 (24.3%)	
Former smoker	332 (31.9%)	200 (30.1%)	132 (35.0%)		974 (35.6%)	582 (35.7%)	392 (35.4%)	
Current smoker	384 (36.9%)	256 (38.6)	128 (33.9%)		970 (35.4%)	524 (32.1%)	446 (40.3%)	
Smoking status (female)				0.829				0.027
No smoker	334 (91.3%)	217 (91.2%)	117 (91.4%)		1070 (81.4%)	499 (73.4%)	571 (90.1%)	
Former smoker	14 (3.8%)	10 (4.2%)	4 (3.1%)		175 (13.3%)	145 (21.3%)	30 (4.7%)	
Current smoker	18 (4.9%)	11 (4.6%)	7 (5.5%)		69 (5.3%)	36 (5.3%)	33 (5.2%)	
Family history of cardiovascular disease	111 (7.9%)	83 (5.9%)	28 (5.5%)	0.015	327 (8.1%)	175 (7.7%)	126 (7.0%)	0.398
History of myocardial infarction	142 (10.1%)	84 (9.2%)	49 (9.7%)	0.717	458 (11.3%)	262 (11.6%)	235 (13.1%)	0.138
Chronic angina	160 (11.4%)	93 (10.3%)	46 (9.1%)	0.045	388 (9.6%)	256 (11.3%)	183 (10.2%)	0.263
History of heart failure	43 (3.1%)	23 (2.5%)	20 (4.0%)	0.140	279 (6.9%)	101 (4.5%)	163 (9.1%)	** < 0.0001**
Chronic lung disease	40 (2.8%)	31 (3.4%)	9 (1.8%)	0.073	220 (5.4%)	107 (4.7%)	118 (6.6%)	0.010
Chronic renal disease*	107 (7.6%)	45 (5.0%)	62 (12.3%)	** < 0.0001**	338 (8.3%)	156 (6.9%)	211 (11.8%)	** < 0.0001**
Peripheral vascular disease	73 (5.2%)	45 (5.0%)	28 (5.5%)	0.652	225 (5.6%)	126 (5.6%)	112 (6.3%)	0.358
**Clinical presentation and examination**								
Heartrate*	83.78 ± 25.27	80.54 ± 23.79	89.56 ± 26.78	** < 0.0001**	86.54 ± 25.66	81.84 ± 23.31	93.56 ± 28.39	** < 0.0001**
Systolic blood pressure*	132.06 ± 31.25	137.70 ± 30.23	121.97 ± 30.52	** < 0.0001**	113.86 ± 50.27	135.08 ± 30.22	121.70 ± 32.70	** < 0.0001**
Diastolic blood pressure*	78.20 ± 42.91	81.25 ± 51.23	72.75 ± 20.00	** < 0.0001**	67.28 ± 32.66	77.30 ± 18.15	73.60 ± 30.54	** < 0.0001**
Killip classification*				** < 0.0001**				** < 0.0001**
Killip I	720 (51.2%)	570 (63.2%)	150 (29.7%)		1770 (43.7%)	1347 (59.5%)	424 (23.6%)	
Killip II	321 (22.8%)	213 (23.6%)	108 (21.3%)		983 (24.3%)	512 (22.6%)	471 (26.3%)	
Killip III	111 (7.9%)	47 (5.2%)	64 (12.7%)		315 (7.8%)	116 (5.1%)	199 (11.1%)	
Killip IV	255 (18.1%)	72 (8.0%)	183 (36.2%)		985 (24.3%)	287 (12.7%)	698 (39.0%)	
**Baseline investigation**								
Total cholesterol (TC)	4.89 ± 1.41	5.06 ± 1.31	4.58 ± 1.52	**0.013**	4.86 ± 8.75	5.25 ± 11.23	4.82 ± 3.60	**0.086**
HD-C*	1.15 ± 0.34	1.17 ± 0.34	1.10 ± 0.34	** < 0.0001**	1.25 ± 3.83	1.28 ± 5.10	1.10 ± 3.54	**0.106**
LD-C*	3.10 ± 1.39	3.23 ± 1.42	2.84 ± 1.30	** < 0.0001**	4.41 ± 25.14	3.24 ± 4.91	4.52 ± 37.39	**0.150**
Triglyceride	1.52 ± 0.89	1.56 ± 0.99	1.45 ± 0.68	0.026	1.65 ± 5.21	1.48 ± 0.87	1.87 ± 7.78	0.134
Fasting blood sugar*	9.59 ± 5.31	8.15 ± 3.97	12.02 ± 6.21	** < 0.0001**	8.87 ± 7.66	8.53 ± 5.50	11.1 ± 8.90	** < 0.0001**
ECG findings								
ST elevation ≥ 1 mm	630 (44.8%)	412 (45.7%)	218 (43.2%)	0.364	1628 (40.2%)	1047 (46.3%)	581 (32.43%)	0.003
ST elevation ≥ 2 mm	807 (42,6%)	508 (56.3%)	299 (59.4%)	0.293	2102 (51.9%)	1258 (55.6%)	844 (47.12%)	0.019
ST depression ≥ 0.5 mm	145 (10.9%)	94 (10.4%)	60 (11.9%)	0.400	464 (11.4%)	268 (11.8%)	196 (10.94%)	0.243
T-ware inversion ≥ 1 mm	89 (6.3%)	71 (7.9%)	18 (3.6%)	0.001	261 (6.4%)	172 (7.6%)	89 (4.97%)	< 0.0001
Bundle branch block	79 (5.6%)	30 (3.3%)	49 (9.7%)	** < 0.0001**	409 (10.1%)	73 (3.2%)	336 (18.76%)	< 0.0001
Non-specific	46 (3.3%)	40 (4.4%)	6 (1.2%)	0.001	1869 (46.1%)	950 (42.0%)	919 (51.31%)	0.689
Infarct location								
Inferior leads	671 (47.7%)	459 (50.9%)	212 (42.0%)	0.001	2182 (53.8%)	1190 (52.6%)	992 (55.39%)	< 0.0001
Anterior leads	722 (51.3%)	433 (48.0%)	289 (57.2%)	0.001	837 (20.7%)	562 (24.8%)	275 (15.35%)	0.596
Lateral leads	355 (25.2%)	234 (25.9%)	121 (24.0%)	0.412	330 (8.1%)	296 (13.1%)	34 (1.90%)	0.0001
True posterior	150 (10.7%)	86 (9.5%)	64 (12.7%)	0.067	320 (7.9%)	169 (7.5%)	151 (8.43%)	0.277
Right ventricle	113 (8.0%)	72 (8.0%)	41 (8.1%)	0.798	343 (8.46%)	259 (11.6%)	84 (4.69%)	0.0001
None	68 (4.8%)	65 (7.2%)	3 (0.6%)	0.205	203 (5.0%)	112 (4.9%)	91 (5.08%)	0.0001
**Invasive therapeutic procedures**								
Cardiac catheterization*	582 (41.4%)	421 (46.7%)	161 (31.9%)	** < 0.0001**	1306 (32.2%)	754 (33.3%)	552 (30.82%)	** < 0.0001**
PCI	412 (29.3%)	283 (31.4%)	129 (25.5%)	0.021	1197 (29.5%)	779 (34.4%)	418 (23.34%)	** < 0.0001**
FB status (expand)	947 (67.3%)	576 (63.9%)	371 (73.5%)	0.038	1897 (71.5%)	1381 (61.1%)	516 (28.81%)	** < 0.0001**
**Pharmacological therapy**								
ASA*	1349 (95.9%)	883 (97.9%)	466 (92.3%)	** < 0.0001**	3482 (85.9%)	2204 (97.4%)	1278 (71.36%)	** < 0.0001**
GP receptor inhibitor	27 (1.9%)	19 (2.1%)	8 (1.6%)	0.493	257 (6.3%)	62 (2.7%)	195 (10.89%)	** < 0.0001**
Unfractionated heparin	226 (16.1%)	147 (16.3%)	79 (15.6%)	0.749	789 (19.5%)	332 (14.7%)	457 (25.52%)	0.616
LMWH	396 (28.1%)	240 (26.6.%)	156 (30.9%)	0.087	1497 (36.9%)	659 (29.1%)	838 (46.79%)	** < 0.0001**
Beta blockers*	708 (50.3%)	577 (64.0%)	131 (25.9%)	** < 0.0001**	1546 (38.1%)	1291 (57.1%)	255 (14.24%)	** < 0.0001**
ACE inhibitor*	566 (40.2%)	453 (50.2%)	113 (22.4%)	** < 0.0001**	1844 (45.5%)	1141 (50.4%)	703 (39.25%)	** < 0.0001**
Angiotensin II receptor blocker	37 (2.6%)	31 (2.9%)	6 (1.2%)	0.011	149 (3.7%)	95 (4.2%)	54 (3.02%)	0.265
Statin	1322 (94.0%)	862 (95.6%)	460 (91.1%)	0.002	3236 (79.8%)	2120 (93.7%)	1116 (62.31%)	** < 0.0001**
Lipid	23 (1.6%)	20 (2.2%)	3 (0.6%)	0.021	132 (3.3%)	51 (2.3%)	81 (4.52%)	0.069
Diuretics*	505 (35.9%)	281 (31.2%)	224 (44.4%)	** < 0.0001**	1247 (30.8%)	652 (28.8%)	595 (33.22%)	** < 0.0001**
Calcium antagonist	95 (6.8%)	70 (7.8%)	25 (5.0%)	0.044	401 (9.9%)	187 (8.3%)	214 (11.95%)	0.127
Oral hypoglycaemic agent*	234 (16.6%)	199 (22.1%)	35 (6.9%)	** < 0.0001**	496 (12.2%)	412 (18.2%)	84 (4.69%)	** < 0.0001**
Insulin*	411 (29.2%)	212 (23.5%)	199 (39.4%)	** < 0.0001**	981 (24.2%)	540 (23.9%)	**441 (24.62%)**	** < 0.0001**
Anti-arrhythmic agent*	97 (6.9%)	43 (4.8%)	54 (10.7%)	** < 0.0001**	436 (10.8%)	159 (7.0%)	277 (15.47%)	** < 0.0001**

*CAD* coronary artery disease, *HDL* high-density lipoprotein, *LDL* low-density lipoprotein, *ECG* electrocardiogram, *PCI* percutaneous coronary intervention, *CABG* coronary artery bypass graft, *ASA* acetylsalicylic acid (aspirin), *GP* glycoprotein, *LMWH* low molecular-weight heparin, *ACE* Angiotensin-converting enzyme.

The asterisk (*) with p-value < 0.0001 indicated that the variable difference between the alive and dead group is statistically significant.

Significant values are given in bold.

Table 2 also demonstrates the imputed data’s summary statistics. The dataset was imputed using the predictive mean matching method. The imputed datasets on elderly patients have an average age of 73 years. In the imputed dataset, the overall mortality rate is 44.83%. There was a significant difference between survival and non-survival in age, gender, hypertension, diabetes, history of heart failure, chronic renal disease, heart rate, systolic blood pressure, diastolic blood pressure, Killip classification, fasting blood sugar, t-wave inversion ≥ 1 mm, bundle branch block bundle, ECG abnormal in inferior leads and anterior leads, cardiac catheterization, PCI, aspirin, GPRI, LMWH, beta-blocker, ACE inhibitor, statin, diuretics, oral hypoglycaemic agent, insulin, and Anti-arrhythmic agent (all variables with p-value < 0.0001).

### Algorithm performance on complete cases

Table 3 illustrates model performances developed in this study. ML models constructed using reduced sets of features demonstrated higher performance compared to ML models developed using a complete set of features LR (0.91 vs 0.83), RF (0.91 vs 0.89), XGB (0.89 vs 0.89) and SVM (0.91 vs 0.87). XGB automatically selects the most important variable^40^ in prediction when using a complete set of variables, a similar AUC of (0.89) was reported after using a reduced set of variables. ML models RF (varImp-SBE-RF) (0.91), SVM (varImp-SBE-SVM) (0.91) and LR (varImp-SBE-LR) (0.91) constructed using selected features performed similarly and comparison was non-significant. However as illustrated in Table 2, DL (all features) model (0.93) using a complete set of features performed slightly better than ML models constructed using a reduced set of features RF (varImp-SBE-RF) (vs. 0.91, p < 0.0001), LR (varImp-SBE-LR) (vs. 0.91, p < 0.0001) and SVM (varImp-SBE-SVM) (vs. 0.91, p = 0.309).

**Table 3:** The AUC DL and ML models with and without feature selection based on a 30% validation dataset.

Models	AUC (95% CI)	Accuracy (95% CI)	Sensitivity	Specificity	PPV	NPV	Kappa value	Mcnemar’s test (p-value)
LR (all)	0.831 (0.762–0.901)	0.832 (0.795,0.865)	0.640	0.855	0.348	0.952	0.362	< 0.001
LR (varImp-SBE-LR)	0.907 (0.867–0.946)	0.809 (0.770,0.843)	0.820	0.807	0.339	0.974	0.386	< 0.001
RF (all)	0.892 (0.849–0.935)	0.908 (0.878,0.932)	0.560	0.949	0.571	0.947	0.514	1.000
RF (varImp-SBE-RF)	0.914 (0.871–0.956)	0.914 (0.885,0.938)	0.640	0.947	0.593	0.956	0.567	0.635
SVM (all)	0.871 (0.814–0.928)	0.901 (0.870, 0.927)	0.600	0.937	0.536	0.951	0.510	0.4610
SVM (varImp-SBE-SVM)	0.911 (0.877–0.946)	0.819 (0.781,0.853)	0.880	0.812	0.361	0.983	0.424	< 0.001
XGBoost (all)	0.894 (0.852–0.936)	0.890 (0.858, 0.917)	0.580	0.928	0.492	0.948	0.471	0.263
XGBoost (varImp-SBE-XGBoost)	0.891 (0.849–0.933)	0.871 (0.837,0.900)	0.680	0.894	0.436	0.959	0.461	< 0.001
DL (all)	0.927 (0.907–0.941)	0,871 (0.837,0.900)	0.680	0.894	0.436	0.959	0.461	< 0.001
DL (LR selected var)	0.941 (0.927–0.955)	0.839 (0.802, 0.871)	0.680	0.857	0.366	0.957	0.390	< 0.001
DL (RF selected var)	0.954 (0.942–0.966)	0.867 (0.832, 0.896)	0.760	0.880	0.432	0.968	0.479	< 0.001
DL (SVM selected var)	0.939 (0.925–0.952)	0.850 (0.814, 0,881)	0.800	0.855	0.400	0.973	0.455	< 0.001
DL (XGBoost selected var)	0.937 (0.923–0.951)	0.817 (0.779, 0.851)	0.720	0.829	0.336	0.961	0.366	< 0.001

Slightly lower AUC value were observed with DL (all features) model using complete set of features (AUC = 0.93) compared to DL models constructed using selected features from DL (RF selected var) (vs. 0.95, p < 0.0001) using 13 predictors, DL (XGB selected var) (vs. 0.94, p < 0.0001) using 6 predictors, DL (SVM selected var) (vs. 0.94, p < 0.0001) with 11 predictors and DL (LR selected var) (vs. 0.94, p < 0.0001) using 15 predictors. There was no statistical significance between all the DL models constructed using selected features from ML (p > 0.05).

Theoretically, by running a model to indicate survival for a new patient aged 65 years and above after STEMI, in the DL (XGB selected var) model with the reduced 6 features selected from XGB, the average mortality risk is reduced to 4% (NPV). While the model is to indicate non-survival, the average risk of a patient being decreased is increased to 37% (PPV). This corresponds to an average 9.25% risk ratio for the outcome in patients classified as non-survival versus survival. Meanwhile, for the DL (RF selected var) model with the reduced features from RF (13 features), the average mortality risk is reduced to 3.2% (NPV). While the model is to indicate non-survival, the average risk of a patient being deceased is increased to 43% (PPV). This corresponds to an average 13% risk ratio for the outcome in patients classified as non-survival versus survival.

### Model prediction using the imputed dataset

The best DL models, DL (RF selected var) and DL (XGB selected var) were also trained on an imputed dataset and tested using a complete case validation dataset. This allows for a valid comparison of models built with imputed and complete case models. Best models trained on imputed datasets performed comparably to models trained on complete dataset on similar validation datasets of complete cases: DL (RF selected var) (AUC = 0.956 (0.944–0.968) vs AUC = 0.954 (0.942–0.966), p = 0.540) and DL (XGB selected var (AUC = 0.948 (0.935–0.960) vs AUC = 0.937 (0.923–0.951) p < 0.0001). There is no statistically significant difference between the DL model (RF selected var) using complete cases with the imputed model.

### Feature selection

Table 4 displays the variables chosen by combining SBE and ML algorithm feature selection methods, which resulted in the ML model with the best predictive performance while using the minimum varaibles. Patient age, fasting blood glucose, heart rate, Killip class, oral hypoglycemic agent, systolic blood pressure, and total cholesterol are all common predictors across best ML models. These predictors were also identified as significant predictors in univariate analysis. The XGB model chose the fewest predictors (six): patient age, fasting blood glucose, heart rate, Killip class, and beta-blocker. Age, Killip Class, and Systolic Blood Pressure are similar features selected by ML feature selection with TIMI risk score.

**Table 4:** Predictors of best ML models.

	ML algorithm				TIMI
	LR	RF	SVM	XGB	
Abnormal BBB in ECG	*				*
ACE inhibitors	*				
Age	*	*	*	*	*
Anti-arrhythmic agent					
ASA					
Beta-blocker	*	*		*	
Cardiac catheterization		*	*		
CRD					
Diabetes mellitus			*		*
Diastolic blood pressure		*	*		*
Diuretics					
ECG abnormal at lateral leads	*				
Ethics					
Family history of CVD		*			
Fasting blood glucose	*	*	*	*	
HDLC		*			
Heart rate	*	*	*	*	
History of chronic renal disease	*				
Insulin					
Killip class	*	*	*	*	*
LDLC					
LMWH	*	*			
Oral hypoglycemia agent	*	*	*		
PCI	*		*		
Race	*				
Systolic blood pressure	*	*	*	*	*
Total cholesterol	*	*	*		

### Comparison with TIMI conventional risk score

**Figure 2:**
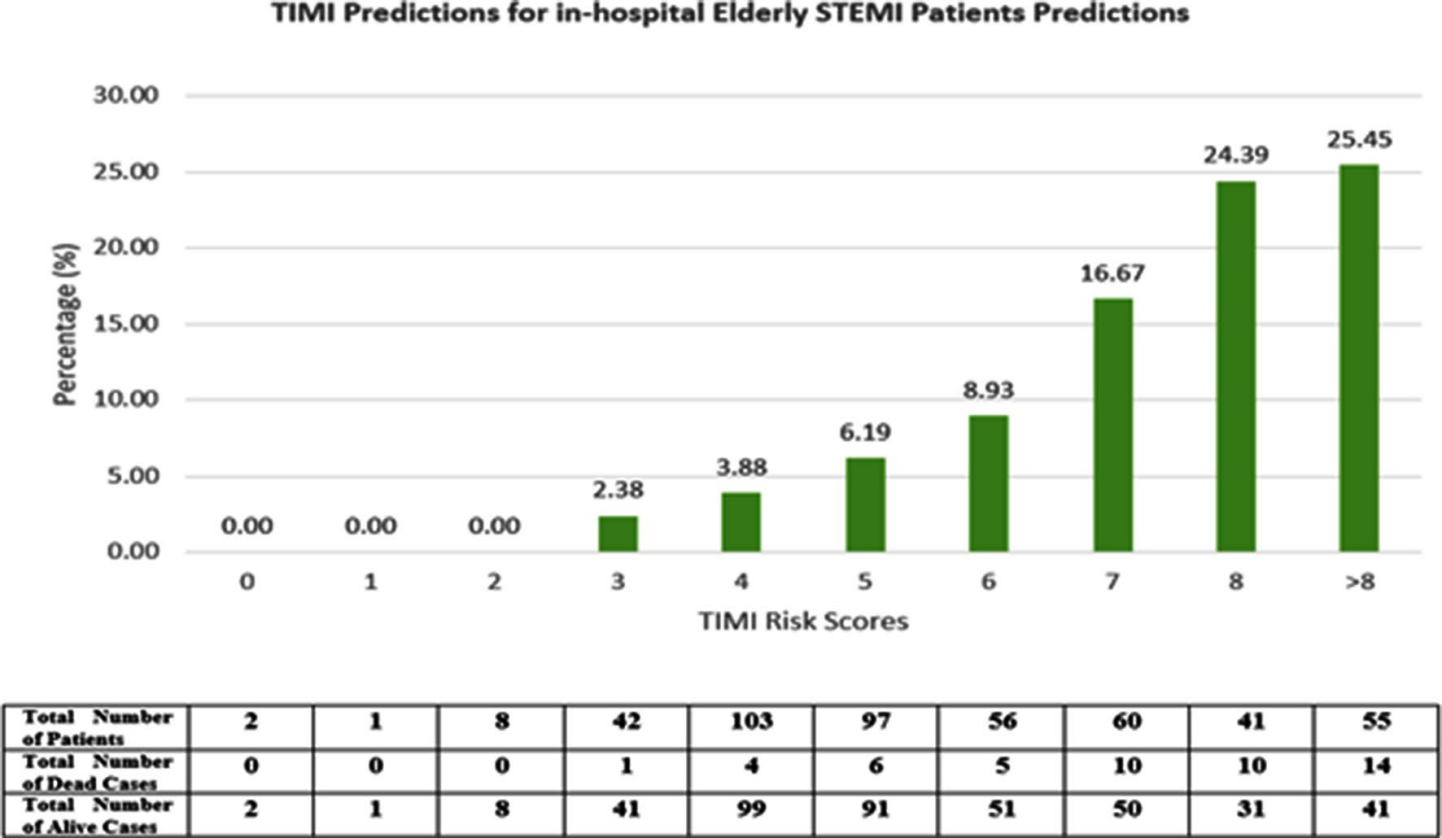
Mortality rate distribution on the validation set of TIMI risk scores.

**Figure 3:**
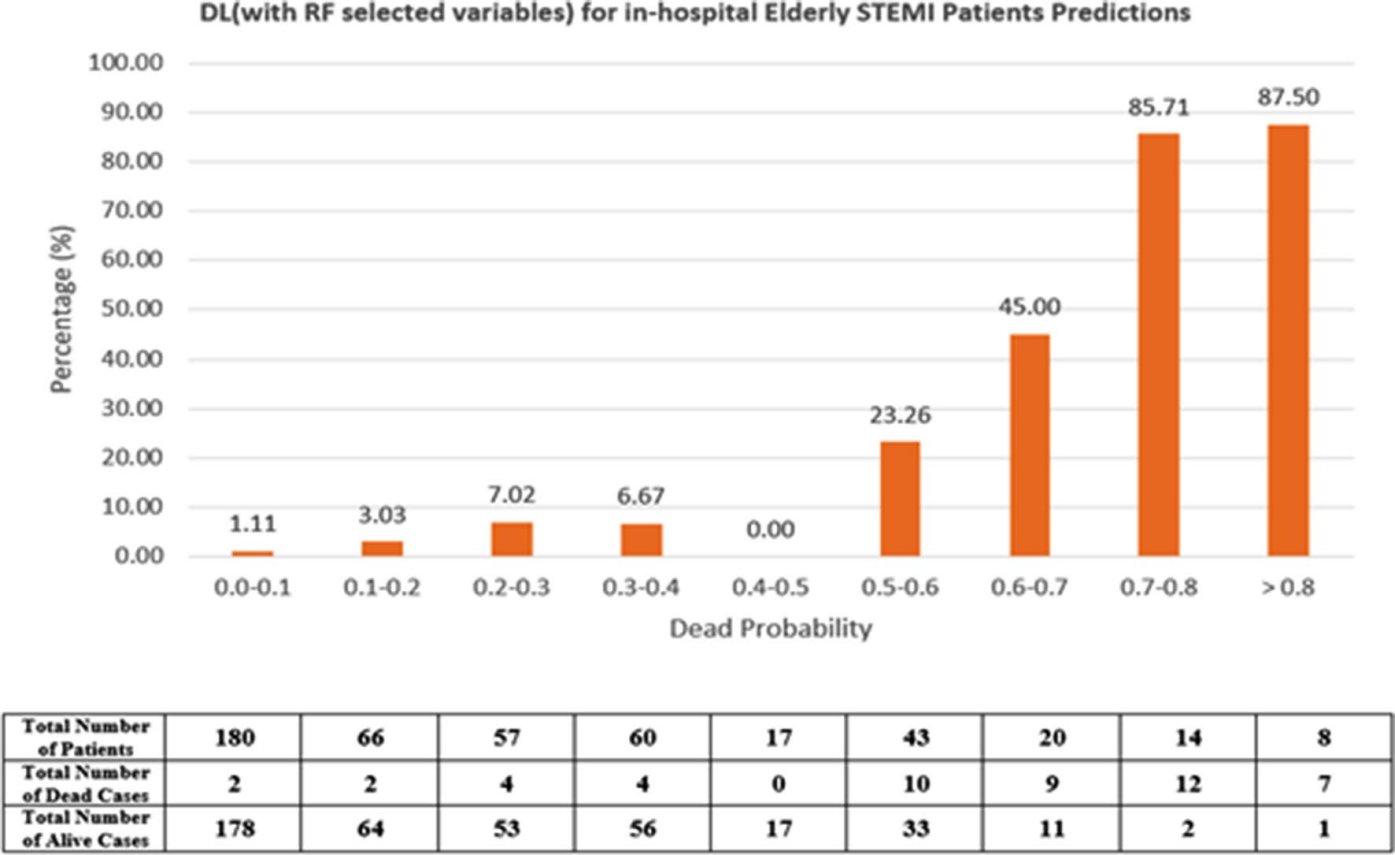
Mortality rate distribution on the validation set of DL (using RF variables) model.

**Figure 4:**
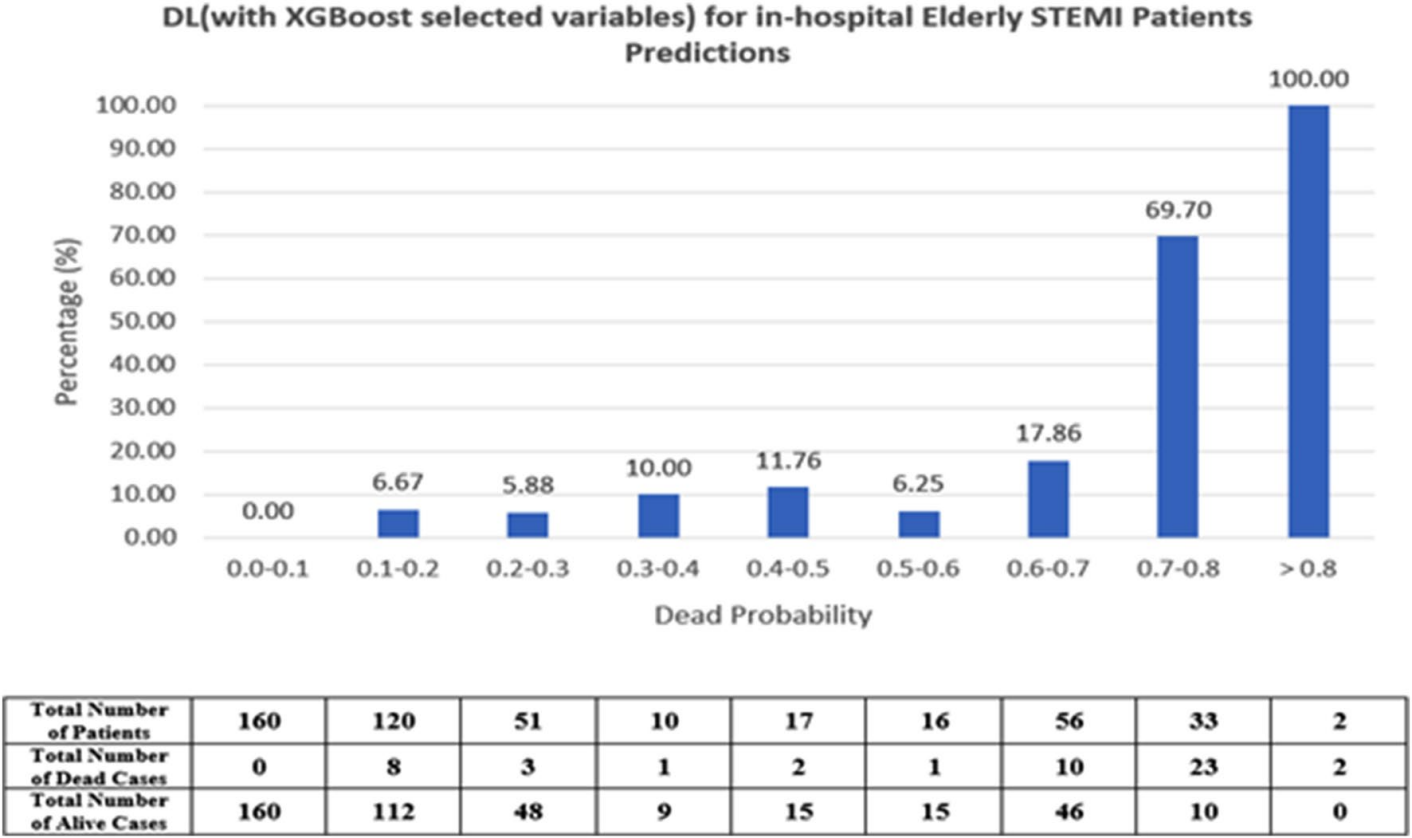
Mortality rate distribution on the validation set of DL (using XGBoost variables) model.

Using the same validation set, TIMI achieved a lower AUC of 0.750 (95% CI 0.669,0.810) compared to all ML and DL models. Figures 2, 3, and 4 illustrate the graph plotted from the TIMI risk score, DL (RF selected var), and DL (XGB selected variables) in predicting the mortality risk of the elderly STEMI patients respectively. For the elderly patients, the ML score categorized patients as low risk with the probability of < 40% and high-risk stratum as ≥ 40%. This is equivalent to a TIMI low-risk of score ≤ 5 and a high-risk score of > 5^43^.

Table 5 tabulates the percentage of mortality in the patients with predicted low risk (TIMI score: < 5; ML probabilities < 0.4) and high risk (TIMI score: > 5; ML probabilities: ≥ 0.4). In the high-risk group, ML and DL predicted mortality better in comparison to TIMI for in-hospital death in elderly patients.

**Table 5:** Percentage of mortality of TIMI score (> 5) and DL-based on risk stratification (> 0.4).

Model	High-risk patients mortality (%)
TIMI risk scores	18.4
DL (with RF selected variables)	44.71
DL (with XGBoost selected variables)	33.64

### NRI analysis

NRI for the in-hospital model, the net reclassification of elderly STEMI patients using the DL (SVM selected var) (Table 6) and DL (XGB selected var) (Table 7) produced a net reclassification improvement of 18.14% with p < 0.00001 over the original TIMI risk score.

**Table 6:** NRI analysis for TIMI vs DL (with RF selected variables).

In-hospital elderly						
		Number of individuals		Reclassification		Net correctly reclassified (%)
		Deep learning		Increased risk	Decreased risk	
		Low risk	High risk			
**Individuals with events (died) (n = 50)**						
	TIMI score			6	9	− 3/50 = − 6.00%
	Low risk	5	6			
	High risk	9	30			
**Individuals without events (alive (n = 415)**						
	TIMI score			14	116	102/415 = 24.58%
	Low risk	228	14			
	High risk	116	57			
Net Reclassification Index (NRI)	− 6.00 + 24.58 = 18.58					
Z, p-value	Z = $\frac{18.58}{\sqrt{\frac{6+9}{{50}^{2}}+\frac{14+116 }{{415}^{2}}}}$ = 226.07 226.07, p < 0.00001					
Conclusion	It was statistically significant. The predictive power of the DL model was improved as compared to the TIMI Risk Scores Model in predicting the mortality rate of elderly STEMI patients, and the proportion of correct classification increased by 18.58%

**Table 7:** NRI analysis TIMI vs DL (with XGB selected variables).

In-hospital elderly						
		Number of individuals		Reclassification		Net correctly reclassified (%)
		Deep learning		Increased risk	Decreased risk	
		Low risk	High risk			
**Individuals with events (died) (n = 50)**						
	TIMI score			6	9	− 3/50 = − 6.00%
	Low risk	5	6			
	High risk	9	30			
**Individuals without events (alive) (n = 415)**						
	TIMI score			14	116	102/415 = 24.58%
	Low Risk	228	14			
	High Risk	116	57			
Net Reclassificaton Index (NRI)	− 6.00 + 24.58 = 18.58					
Z, p-value	Z = $\frac{18.58}{\sqrt{\frac{6+9}{{50}^{2}}+\frac{14+116 }{{415}^{2}}}}$ = 226.07 226.07, p < 0.00001					
Conclusion	It was statistically significant. The predictive power of the DL model was improved as compared to the TIMI Risk Scores Model in predicting the mortality rate of elderly STEMI patients, and the proportion of correct classification increased by 18.58%					

## Discussion

This study aimed to construct and validate conventional ML and DL models in Asian elderly admitted with STEMI. We also compared the predictive performance of these models against conventional risk score models such as TIMI. This is the first study to include DL and conventional ML models in the risk prediction of in-hospital mortality in Asian elderly with STEMI resulting in a higher predictive ability than the conventional statistical method (TIMI). DL and ML risk stratification models were developed based on the Asian elderly on relatively recent data, which can better predict mortality for STEMI patients in the current practice compared to TIMI.

We observed from the results obtained in this study that (i) DL model (AUC = 0.93) outperform all ML models (AUC ranging from 0.83 to 0.89) on a complete set of features (p < 0.0001) (ii) DL models constructed using ML feature selection (AUC ranging from 0.93 to 0.95) performed better than ML constructed using selected features (AUC ranging from 0.89 to 0.91) (p < 0.0001) (iii) Both DL and ML model constructed using all and selected features (AUC ranging from 0.83 to 0.95) outperformed conventional risk scoring score TIMI (AUC = 0.75) (iv). DL constructed using selected features (AUC ranging from 0.93 to 0.95) were observed to perform better than DL constructed using all features (AUC = 0.93). DL is composed of multiple feature processing layers obtained by composing simple but nonlinear modules, each of which transforms a feature at one level into a feature at a higher, slightly more abstract level^13,45^. As a result, when compared to ML and the conventional method TIMI score, the higher accuracy obtained with DL in this study is due to the algorithm discrimination power and features used. This is supported by Kwon's findings^13^, which show that DL outperforms ML and conventional risk scores in predicting mortality in Korean ACS patients.

These risk-scoring models are developed using logistic regression with the limitation of predetermined expectations on data behaviour, and preselected parameters in the development phase^13^. Further limitations include a lack of bedside convenience and some data only being available following a biochemical test. Since age is a component of risk stratification in-hospital mortality is significantly higher in older adults. As age is incorporated into most conventional risk score algorithms older adults will be scored as higher risk based on their age alone^46^. Several previous studies on mortality prediction also have reported on the use of feature selection techniques to enhance the performance of machine learning algorithms by reducing the predictor's dimensionality in Asian patients. This study also demonstrated that ML-based models outperformed conventional risk score TIMI^18,30,47,48^.

Additionally, previous research has also shown that models based on DL perform better in classification tasks than models based on classical ML algorithms and conventional risk scores^13^. Similar findings were reported in our study as well.

Even though the TIMI risk score has been widely used in the Asian population, this score was developed from the Western Caucasian cohort with limited data from an Asian population. In our study, when DL and ML models were validated against TIMI, we observed a modest AUC value of 0.75 for TIMI score validated on elderly Asian patients which were lower than the TIMI risk score reported on in a fibrinolytic eligible STEMI population AUC of 0.78^49^. Modest performance AUC of 0.709 (95% CI 0.591–0.827; p < 0.001) have also been reported on TIMI risk score for in-hospital mortality of older women age > 70 who underwent PPCI in a South Asian country^50^.

We also conducted an accuracy test using data that were not used for the model derivation for comparison with TIMI. We used two DL models as there was no significant difference between DL models constructed using selected variables. Hence, the two DL models used were; the DL (RF selected var) model with the highest performance (AUC = 0.95) with 13 predictors and the DL (XGB selected variable) (AUC = 0.93) with the least number of predictors^6^. Both algorithms make use of decision trees, while XGB makes use of boosting rather than bagging. This approach reduces variance and bias^32^. Numerous recent investigations have demonstrated the generalizability and robustness of both methods in clinical practice. Both models managed to identify high-risk patients that reported higher mortality in those classified as high risk in TIMI. The mortality rate, however, was no different suggesting an inherent inaccuracy within the algorithm. The mortality for high-risk patients for TIMI in this study is 18% vs 44% for DL (RF selected variable) model.

The TIMI risk score lacks risk factors relevant to older adults and fails to account for the overall complexity of the older adult with ACS^13,51^. The Asian cohort was found to be carrying an overall higher disease burden and risk compared to the TIMI cohort. The lack of weighting for the risk factors, while improving usability, decreased TIMI risk score discriminatory performance^52^^,^^53^. Not only that, TIMI is known to underestimate mortality risk in the high-risk group as seen in this study. This may delay proper treatment and sufficient resource allocation to high-risk elderly patients incurring excess avoidable deaths.

It is essential that the risk prediction model be interpretable. To this end, it is true that one of the significant advantages of a deep learning algorithm is its intrinsic hierarchical feature selection along with successive levels of increasing abstraction for pattern detection. While the newly extracted features are largely meaningless from the perspective of the deep learning method, their extraction can be beneficial for driving the learning process in certain circumstances. This was likewise the case in our instance where the DL model with selected features performed similarly or better than DL constructed using all features. Not only that, but a new genre of literature is forming that recounts similar circumstances, such as those found in^54,55^.

Exploring the feasibility of DL and ML on the predictors of mortality among Asian elderly provides clinicians with a tool that allows the identification of higher-risk populations in the emergency department that could influence effective management based on their prognostic characteristics as described by their risk scores. ML methods discussed in this study are needed to rank and select significant risk factors associated with in-hospital mortality of the elderly. Feature selection allows better interpretation of the models by restricting the scope of predictors used, selecting only those clinically relevant, and ease of implementation of the model for bedside risk assessment usage.

Hence, our data-driven model for risk prediction and identification of factors associated with in-hospital mortality was developed using a nationwide registry of a multiethnic Asian elderly population. We identified age, fasting blood glucose, heart rate, Killip class, oral hypoglycemic medication, systolic blood pressure, and total cholesterol to be common predictors of in-hospital mortality in Asian elderly patients following STEMI. Additionally, invasive procedures such as heart catheterization were also selected in our study. These factors are consistent with the findings of this study's univariate analysis. These factors have also been chosen by machine learning and deep learning studies aimed at predicting mortality post STEMI in the Asian population^13,30^. We discovered that STEMI-related treatments have no effect on outcomes in different groups. In the main dataset of STEMI in-hospital patients, 97.3% (16,829) received ASA, while 6176 (35.7%) underwent PCI^18^. In the elderly patient dataset, 3482 patients were given ASA, accounting for 85.9%, and 1197 patients were given PCI, accounting for 29.5%. In terms of significant analysis performed on raw datasets in both studies, both datasets exhibit similar characteristics and yield similar results.

Additionally, we identified common predictive variables between the conventional risk score TIMI and feature-selected by ml algorithms. These variables include age, Killip class, systolic blood pressure, and fasting blood sugar, which is an indicator of diabetes. These factors also corroborate the findings of the univariate analysis in this study.

Older age and higher Killip class were significant predictors of mortality in Asian patients^12,56^. The elderly, especially those aged equal or greater than 65 years old represents a subgroup of high-risk ACS patients due to the fact that they commonly have other comorbidities^57^. Killip class is also noted to be among the factors that are associated with increased mortality in the elderly. Generally, older patients have a higher incidence of heart-related complications (Killip class II-IV) than younger patients^58^. Killip class selected by ML and univariate analysis conforms with the study by^15^ where Killip class is selected as main predictors by ML algorithm. As the most significant determinant of myocardial oxygen and cardiac workload, heart rate plays a vital role in in-hospital mortality and was also selected^59^.

Diabetes in individuals aged ≥ 65 years has globally become a growing public health burden. The prevalence of diabetes and diabetes-related complications, such as myocardial infarction (MI) and ischemic stroke, is increasing in the older age group. Fasting glucose level is a fundamental element in managing diabetes and both high and low fasting glucose levels are associated with a higher risk of mortality^60,61^. Fasting blood glucose has been selected in our study by all ML features selection methods and our previous published study^18^. Pharmacological treatments such as beta-blockers post-STEMI are also often associated with improved outcomes and significant predictors of STEMI patients^3,62–64^. Oral hypoglycemic agent indicates the presence of diabetes and its use by patients during an ACS event may reflect pre-existing diabetes. Knowing the duration of illness with diabetes may have helped risk prediction better as it has been associated with a higher risk of death in other studies^65^. Nonetheless, oral hypoglycaemic agents were selected as the main predictors of mortality of the elderly in our study^66,67^.

Older age has been found to be predictive of lower use of cardiac catheterization, with significant variation internationally^68^. We have noted a significant difference in survival vs non-survival (p < 0.0001) in our study between older patients that underwent cardiac catheterization procedures. However, we identified only 29% of Asian elderly STEMI patients who have undergone PCI and 44% cardiac catheterization. This is despite the data showing that in‐hospital mortality after percutaneous coronary intervention (PCI) has fallen for all age groups over the past several years. Elderly patients with ACS tend to be undertreated, both invasively and pharmacologically. Invasive treatment seems to yield better outcomes for this group of patients^57^. This is an area that needs improvement to raise the level of care.

Data imputation was performed to ensure the validity of the findings. We tested the results of data imputation on model with the highest AUC in this study DL (RF selected var) and model high AUC and least number of predictors DL (XGB selected var). We used multivariable imputation using chained equations and the predictive mean matching method for data imputation. The multivariable imputation using chained equations and predictive mean matching method used in this study was selected as recommended in a similar study conducted on the Swedish heart registry dataset that resulted in high model performance^20^. Additionally, Solaro^69^ studies observed that miss forests a machine learning data imputation method relative performance varied according to the MCAR data patterns and did not provide a clear advantage. In general, miss forests imputation accuracy and applicability remain unknown.

Data imputation techniques produced models with comparable prediction performance to those developed using complete cases. We first excluded patients with more than 50% missing data because this would necessitate data imputation, which could alter our conclusion. We do not believe this is a constraint on the population, given the dataset is still quite large. Due to the fact that the dataset contained complete data for all follow-up time points, risk calculators for both the DL and TIMI calculators could be generated. However, identifying characteristics associated with the use of complete cases for in-hospital elderly mortality prediction would result in more reliable conclusions. We repeated the experiment using an incomplete dataset and imputed data and obtained comparable findings. However, the imputed model for DL (XGB chosen var) performed slightly better, as the DL technique performs better when datasets with lower feature dimensions and a larger number of datasets are utilized.

The cross-validation and hyperparameter tuning approach used in this study increases the efficacy of the DL and ML algorithms during model construction as it reduces the risk of model over-fitting. Also, the classification performance is highly influenced by data pre-processing and tuning of algorithms^70^.

To ensure the study's reliability, all models were validated using untouched validation data. The DL model performed similarly to models with feature selection when using complete sets of variables collected. This refutes the claim that feature selection leads to the loss of important prognostic information as claimed by Kwon^13^.

### Study limitations

Despite the excluded patient, the number of elderly people over the age of 65 (3991 patients) was large enough to allow for analysis; however, we regard this as a limitation of the study. Several other limitations also exist in this study. Firstly, we could only validate DL and ML models for in-hospital, with a clinical prognostic model TIMI score that was designed for 30 days’ mortality. TIMI score was adopted due to its simplicity and it was developed for short term risk stratification. Parameters to calculate GRACE score were not acquired during patient admission compared to TIMI score. Furthermore, studies by Aragam and Correia^43,71^ reported that both scores show similar discriminatory capacity for STEMI in-hospital death, and the TIMI score had better calibration than GRACE. Hence comparing performance for two risk scores appears redundant. In-hospital bleeding was not captured in the NCVD registry, which is a limitation of the study despite the fact that it is an important factor affecting in-hospital mortality, particularly in the elderly. Both GP receptor inhibitors and ASA are relevant in-hospital antiplatelet drug therapy^72^ that were present in the initial complete variable set used for model development but were not selected by the ML feature selection algorithm. The ML feature selection algorithm selects variables that are significant to the outcome^73^. In this study, we discovered that GP receptor inhibitor is not a significant factor using both the univariate and machine learning methods. The majority of elderly patients are given ASA, but it is not chosen as a significant variable affecting mortality by the all ML feature selection method used in this study. As shown in Table 2, smoking is significantly associated with mortality in elderly patients, and similar findings in STEMI patients indicated that smoking affects mortality^18,74^. However, smoking and gender predominance have no effect on mortality in this cohort. In this cohort, which includes 50% of patients aged 65 and above, former and current smokers are men. Meanwhile, female smokers account for only about 0.022% of current and former smokers of all patients.

Future studies using interpretable DL will be our next area of study. Both DL and ML models rely on representability as opposed to medical knowledge which can lead to bias due to the representativeness of training data. It is still unclear whether DL and ML will consistently perform on real live data sets. Hence, the model needs to be continuously evaluated with real-time patient data which can be easily acquired due to the implementation of the Electronic Health Record System in hospitals. These risk scores could be implemented into the hospital electronic systems for physicians’ use. This might be the scope for future studies, as well as validating this risk score in a registry rather than an administrative database. The study's generalizability is relevant to Asians in general, given the NCVD registry's ethnic make-up of Malay, Chinese, and Indian descendants. It is particularly relevant for Malaysia, Brunei, and Singapore, as well as other Asian countries such as China and India^75^.

## Conclusion

We demonstrated that DL with ML feature selection can be applied in conjunction with conventional risk score methods to improve mortality prediction in Asian elderly patients presenting with STEMI. This knowledge could be used to improve communication and awareness among elderly patients, allowing physicians to make management changes and better manage limited resources.

## Supplementary Information

Supplementary Table 1.

## Data Availability

The data that support the findings of this study are available from the National Heart Association of Malaysia (NHAM) but restrictions apply to the availability of these data, and so are not publicly available. The data belongs to the individual ministry of health universities hospitals and private hospitals that require multiple institutional agreements for data release to third parties hence ethical approval is needed for analysis. Data are however available from NHAM upon request using https://www.malaysianheart.org/?p=contact or email them at secretariat@malaysianheart.org. Any findings from the data need to be reported and permission needs to be obtained from the NHAM committee before publication.
