# Supplementary material for: In-hospital risk stratification algorithm of Asian elderly patients
Source: Sci Rep. 2022 Oct 20;12:17592. doi: 10.1038/s41598-022-18839-9 (PMC9584943; doi:10.1038/s41598-022-18839-9)
Supplement: Supplementary file 1 — Supplementary Table 1. [file 41598_2022_18839_MOESM1_ESM.docx]

**Supplementary Table 1: Variables Missing Rate**

| Features | Number of Missing Data | Missing Rate |
| --- | --- | --- |
| DEMOGRAPHICS | | |
| Patient Age | 0 | 0 |
| Patient Race | 865 | 5.68% |
| Patient Gender | 0 | 0 |
| STATUS BEFORE EVENT | | |
| Smoking Status | 1035 | 7.06% |
| History of Hypertension | 1121 | 7.65% |
| History of Diabetes | 1579 | 10.77% |
| Family History of Premature Cardiovascular Disease | 3818 | 26.05% |
| History of Mycardial Infraction | 2305 | 15.73% |
| Documented CAD | 2675 | 18.25% |
| History of Heart Failure | 1821 | 12.42% |
| Chronic Lung disease | 1822 | 12.43% |
| Chronic Renal disease | 1877 | 12.80% |
| History of Cerebrovascular Disease | 1900 | 12.96% |
| CLINICAL PRESENTATION & EXAMINATION | | |
| Heart Rate (bpm) | 4316 | 29.44% |
| Systolic Blood Pressure (mmHg) | 834 | 5.69% |
| Diastolic Blood Pressure (mmHg) | 353 | 2.41% |
| Killip Classifications | 4026 | 27.47% |
| BASELINE INVESTIGATION (values obtained within 48 hours from admission) | | |
| Total Cholesterol (mmol/L) | 2813 | 19.19% |
| HDL (mmol/L) | 2921 | 19.93% |
| LDL (mmol/L) | 2909 | 19.85% |
| Triglyceride (mmol/L) | 2821 | 19.25% |
| Fasting Blood Glucose (mmol/L) | 3020 | 20.60% |
| ELECTROCARDIOGRAPHY (ECG) | | |
| ST-segment Elevation ≥1mm in ≥ 2 Contiguous Limb Leads | 0 | 0 |
| ST-segment Elevation ≥ 2mm in ≥ 2 Contiguous Frontal Leads | 0 | 0 |
| ST-segment Depression ≥ 0.5mm in ≥ 2 Contiguous Leads | 0 | 0 |
| T-wave inversion ≥1mm | 0 | 0 |
| Bundle Branch Block (BBB) | 0 | 0 |
| Non-specific | 0 | 0 |
| ECG Location inferior leads | 0 | 0 |
| ECG Location anterior leads | 0 | 0 |
| ECG Location lateral leads | 0 | 0 |
| ECG Location true posterior | 0 | 0 |
| ECG Location right ventricle | 0 | 0 |
| ECG Location none | 0 | 0 |
| INVASIVE THERAPEUTIC PROCEDURES | | |
| Cardiac Catherization | 808 | 5.51% |
| Percutaneous Coronary Intervention | 977 | 6.67% |
| PHARMACOLOGICAL THERAPY | | |
| ASA | 348 | 2.37% |
| GP Receptor Inhibitor | 1588 | 10.83% |
| Unfractioned Heparin | 1543 | 10.53% |
| LMWH | 1305 | 8.90% |
| Beta-blocker | 1026 | 6.99% |
| ACE Inhibitor | 1100 | 7.50% |
| Angiotensin II Receptor Blocker | 1545 | 10.54% |
| Statin | 471 | 3.21% |
| Other lipid lowering agent | 1573 | 10.73% |
| Diuretics | 1236 | 8.43% |
| Calcium antagonist | 1454 | 9.92% |
| Oral Hyperglycemia | 1402 | 9.56% |
| Insulin | 1343 | 9.16% |
| Anti-arrhythmic Agent | 1600 | 10.92% |
| OUTCOMES | | |
| Patient Outcome | 788 | 5.38% |
